# Collagen-coated superparamagnetic iron oxide nanoparticles as a sustainable catalyst for spirooxindole synthesis

**DOI:** 10.1038/s41598-022-10102-5

**Published:** 2022-04-12

**Authors:** Shima Ghanbari Azarnier, Maryam Esmkhani, Zahra Dolatkhah, Shahrzad Javanshir

**Affiliations:** Heterocyclic Chemistry Research Laboratory, Department of Chemistry, Iran University of Science and Technology, 16846-13114 Tehran, Iran

**Keywords:** Chemistry, Catalysis

## Abstract

In this work, a novel magnetic organic–inorganic hybrid catalyst was fabricated by encapsulating magnetite@silica (Fe_3_O_4_@SiO_2_) nanoparticles with Isinglass protein collagen (IGPC) using epichlorohydrin (ECH) as a crosslinking agent. Characterization studies of the prepared particles were accomplished by various analytical techniques specifically, Fourier transform infrared (FTIR) analysis, scanning electron microscopy (SEM), transmission electron microscopy (TEM), vibrating sample magnetometry (VSM), energy-dispersive X-ray spectroscopy (EDS), X-ray powder diffraction (XRD), thermogravimetric analysis (TGA), and Brunauer−Emmett−Teller (BET) analysis. The XRD results showed a crystalline and amorphous phase which contribute to magnetite and isinglass respectively. Moreover, the formation of the core/shell structure had been confirmed by TEM images. The synthesized Fe_3_O_4_@SiO_2_/ECH/IG was applied as a bifunctional heterogeneous catalyst in the synthesis of spirooxindole derivatives through the multicomponent reaction of isatin, malononitrile, and C-H acids which demonstrated its excellent catalytic properties. The advantages of this green approach were low catalyst loading, short reaction time, stability, and recyclability for at least four runs.

## Introduction

Nowadays, the use of bifunctional catalysts has become a new field to promote chemical reactions in green, and environmentally friendly pathways and processes. In order to develop new approaches that are more respectful of the environment, catalyst development strategies are now oriented towards polymers of natural origin such as polysaccharides or proteins which come from renewable resources, often biocompatible and also more biodegradable than their synthetic counterparts. Bio-based heterogeneous catalysts, prepared from renewable natural polymers, have received significant attention in recent years due to their substantial advantages such as biodegradability, stability, and recyclability. The combination of nanoparticles and biodegradable polymers can result in nano-biocomposites, which have the potential for various catalytic and environmental applications^1–5^.

Plentiful supports have been commonly used for the immobilization of natural polymers such as silica, resins, silica composites, and magnetic materials among others. Magnetic nanoparticles based on metals such as Cu, Co, Fe, and Ni provide a potent solid support system to immobilize proteins^6–10^, among them, magnetite nanoparticles (Fe_3_O_4_) have remarkable properties such as superparamagnetism, low toxicity, high specific surface area, biocompatibility and easy separation which makes them more interesting for researchers.

One of the most commonly used techniques for the immobilization of proteins is cross-linking. For collagen materials, many crosslinkers such as glutaraldehyde, isocyanates, glyoxal, and carbodiimides, were used^11^.

Natural polymers such as polysaccharides (cellulose, chitosan, chitin, alginate, carrageenan, lignin, fucoidan, etc.) and proteins have been used as catalysts in chemical transformations^12–18^. Coating magnetic particles with natural polymers allows them to use their functional groups for promoting chemical reactions and also easy separation^19,20^.

Based on our interest for turning agricultural and marine waste into value added materials^21–23^, we have used Isinglass (IG), a natural polymer derived from swim bladder of fish with high content of collagen protein, for encapsulating Fe_3_O_4_@SiO_2_ nanoparticles using epichlorohydrin (ECH) as crosslinking agent. The prepared hybrid material named Fe_3_O_4_@SiO_2_/ECH/IG was applied as bifunctional heterogeneous catalyst in the synthesis of spirooxindole derivatives. Such hybrid material based on natural polymer IG with both acidic and basic groups has been shown to be a very effective catalyst in a variety of chemical transformation, including the synthesis of, triazoles^24^, 4*H*-pyran derivatives^25^, and Suzuki coupling^26^. IG contains many amino acids whom their properties and catalytic performances have been demonstrated for many years^27,28^.

Defined as processes allowing at least three reagents to react in a single pot, all of which participate in the structure of the final product, multicomponent reactions make it possible to synthesize highly functionalized compounds. In addition, multicomponent reactions offer rapid access to a wide variety of potential active ingredients. In the same way that a 4-digit code offers 10,000 possibilities, the possible variations for each reagent give access to an impressive number of related compounds. Very useful in combinatorial chemistry, MCRs make it possible to constitute chemical libraries for high throughput screening in the pharmaceutical industry. Several evocative tags are commonly involved in MCRs such as the atomic economy, easy to implement, using very mild reaction conditions, and without recourse to toxic metals, these reactions represent a definite step forward to ideal synthesis^29,30^.

**Figure 1:**
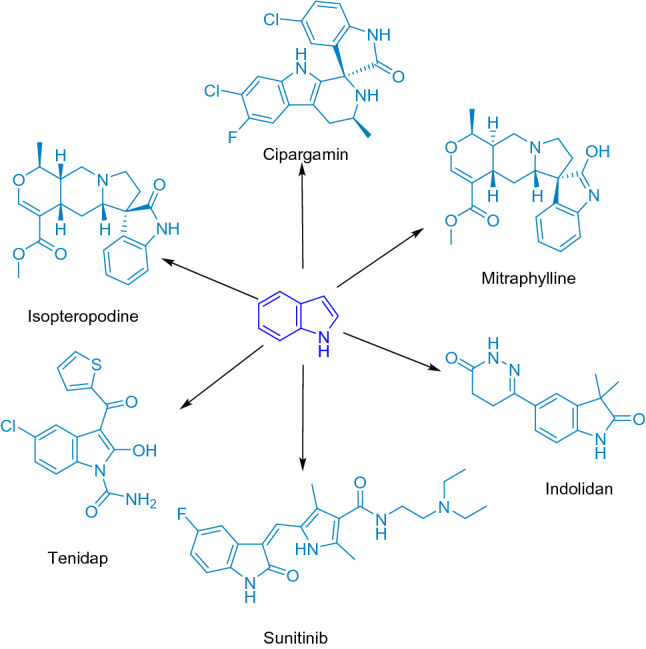
Some biologically active oxindoles and spirooxindoles.

Heterocyclic compounds represent more than 90% of the active ingredients possessing a wide range of applications in pharmaceutical industry, veterinary medicine and phytochemistry^31^. Indoles, oxindoles, and spirooxindoles are important heterocyclic compounds, and ubiquitous motifs in naturally and unnaturally occurring biologically active pharmaceutical ingredients^30^. Some biologically active indoles, oxindoles and spirooxindoles are presented in Fig. 1.

So far, various methods and catalysts have been used for the synthesis of these compounds, including the reaction between isatin, malononitrile and dimedone with different catalysts such as α-amylase^32^, CoFe_2_O_4_@SiO_2_@SO_3_H^33^, MgO@PMO-IL^34^, magnetic sulfonated chitosan^35^, magnetic poly ethyleneimine^36^, etc.

**Figure 2:**
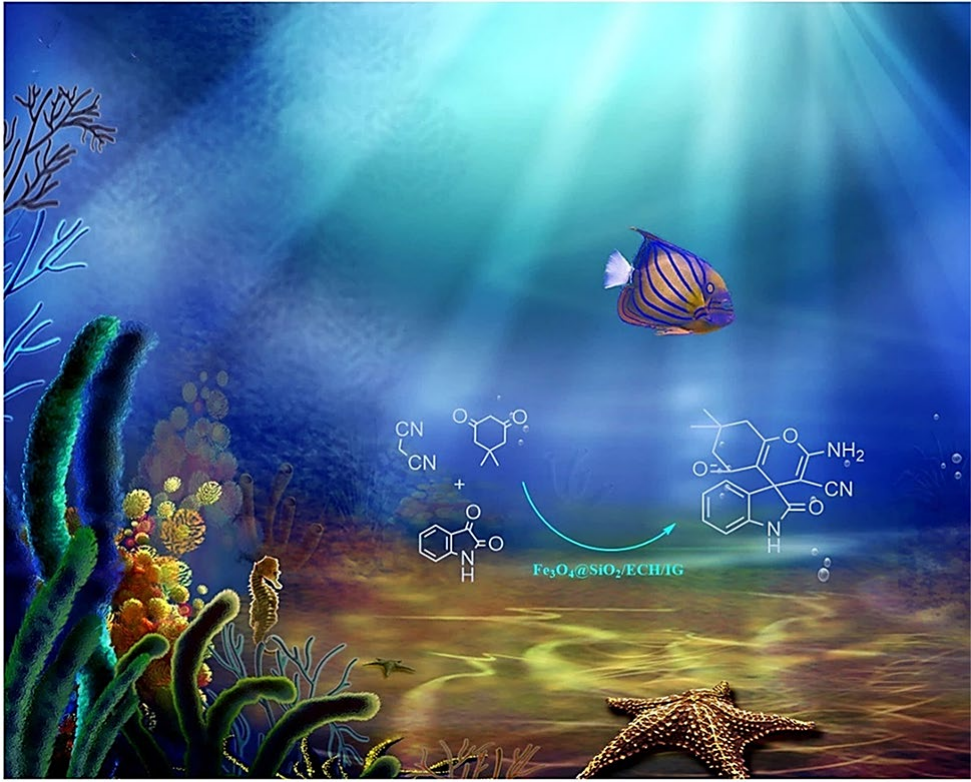
Collagen-coated superparamagnetic iron oxide nanoparticles as a sustainable bio-based catalyst for the direct synthesis of spirooxindole (software used: ChemDraw Ultra 12.0 and Paint 3D).

Here, we have developed a new collagen-coated superparamagnetic iron oxide nanoparticle as a sustainable bio-based catalyst for the direct synthesis of spirooxindole (Fig. 2).

## Results and discussion

**Figure 3:**
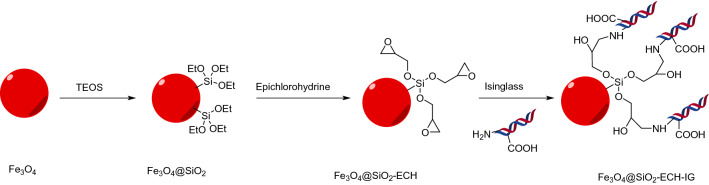
Schematic representation of the catalyst synthesis procedures.

Synthesis pathway of Fe_3_O_4_@SiO_2_/ECH/IG is illustrated in Fig. 3. Fe_3_O_4_ NPs were prepared through a co-precipitation method by dissolving divalent and trivalent iron salts into distilled water, followed by precipitation with NH_4_OH. Afterward, TEOS was hydrolyzed to form silica oligomers, which were coated on the surface of Fe_3_O_4_ nanoparticles to obtain Fe_3_O_4_@SiO_2_ nanoparticles. Subsequently, ECH was cross-linked on the surface of Fe_3_O_4_/SiO_2_. Fe_3_O_4_@SiO_2_/ECH/IG was obtained by nucleophilic addition of IG to as-prepared magnetic nanoparticles.

### Characterization of the Fe_3_O_4_@SiO_2_/ECH/IG

The structure, morphology and magnetic properties of the prepared nanocatalyst were accurately characterized by different analytical techniques. The FT-IR spectra of the Fe_3_O_4_@SiO_2_/ECH/IG, Fe_3_O_4_@SiO_2_, and Fe_3_O_4_ are compared in Fig. S1 (see supporting information). The FTIR spectrum of Fe_3_O_4_ indicates the characteristic band of Fe–O at 596 cm^−1^^37^.

The sharp bands at 1072 cm^−1^ and 816 cm^−1^ were assigned to the asymmetric and symmetric linear stretching vibrations of Si–O–Si bonding respectively. The bending vibration absorption peak of Si–O–Si was also perceived at 464 cm^−1^^38^. The absorption peak at 3426 cm^−1^ which was assigned to the O–H stretching vibrations was shifted from 3426 to 3276 cm^−1^ in Fe_3_O_4_@SiO_2_/ECH/IG with a net diminution of intensity indicating the involvement of isinglass in the synthesis of final composite. The characteristic bands appeared at 1400, 1385 and 1220 cm^-1^ were attributed to C–O (carboxyl), C–OH (secondary) and C–O groups. Finally, this FT-IR spectrum can be clearly shown that the Fe_3_O_4_@SiO_2_/ECH/IG was successfully prepared.

**Figure 4:**
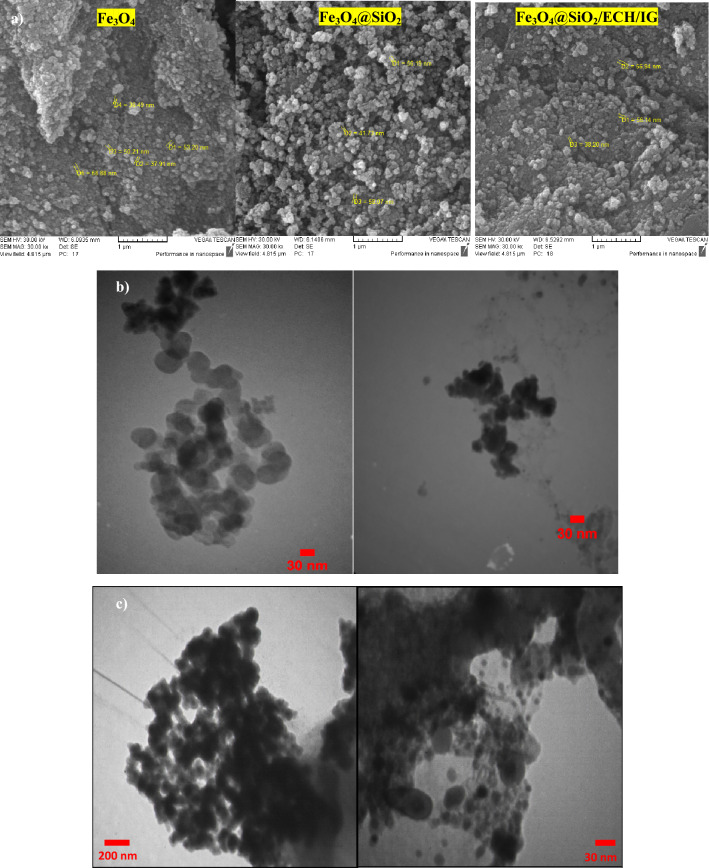
(**a**) SEM images of Fe_3_O_4_, Fe_3_O_4_@SiO_2_ and Fe_3_O_4_@SiO_2_/ECH/IG and (**b**) TEM images of Fe_3_O_4_@SiO_2_/ECH/IG before reaction and (**c**) TEM images of Fe_3_O_4_@SiO_2_/ECH/IG after recycling.

The morphology and the structure of the Fe_3_O_4_@SiO_2_/ECH/IG was characterized by SEM and TEM analysis (Fig. 4a,b,c). The average particle size was estimated to be 58 nm. Moreover, the well-ordered structure of the catalyst and its almost uniform distribution are clearly observable (Fig. 4a). The core–shell structure of the magnetic particles was proofed with the black centres and the brightest areas as Fe_3_O_4_ cores and SiO_2_ shells respectively. These images also approve that the particles are nanometric in size (Fig. 4b). Moreover, the TEM images of the catalyst after the recycling process show that the structure of the nanocatalyst didn’t change during the reaction (Fig. 4c), which provides clear evidence of the stability of the prepared catalyst.

**Figure 5:**
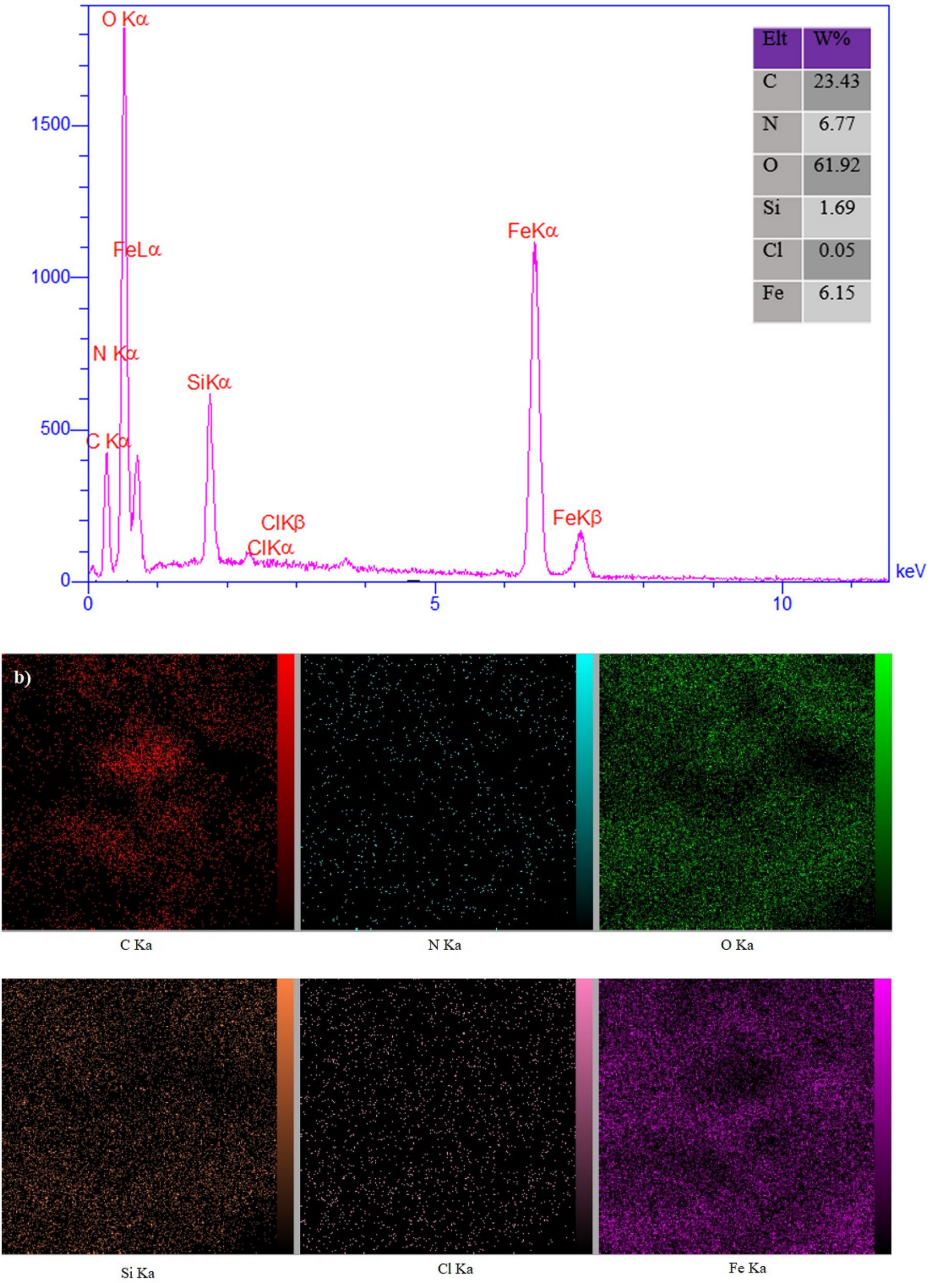
(**a**) Energy dispersive X-ray analysis (EDX) of Fe_3_O_4_@SiO_2_/ECH/IG and (**b**) elemental mapping of C (red); N (blue), O (green), Fe (violet), Si (orange) and Cl (pink) atoms for Fe_3_O_4_@SiO_2_/ECH/IG.

Furthermore, the presence of carbon, oxygen, nitrogen, iron and Si elements (ratios of 23.43: 61.92: 6.77: 6.15: 1.69 wt%, respectively) was confirmed by EDX analysis shown in Fig. 5a and b. It confirms that the incorporation of expected elements into the structure of the prepared catalyst was achieved successfully.

**Figure 6:**
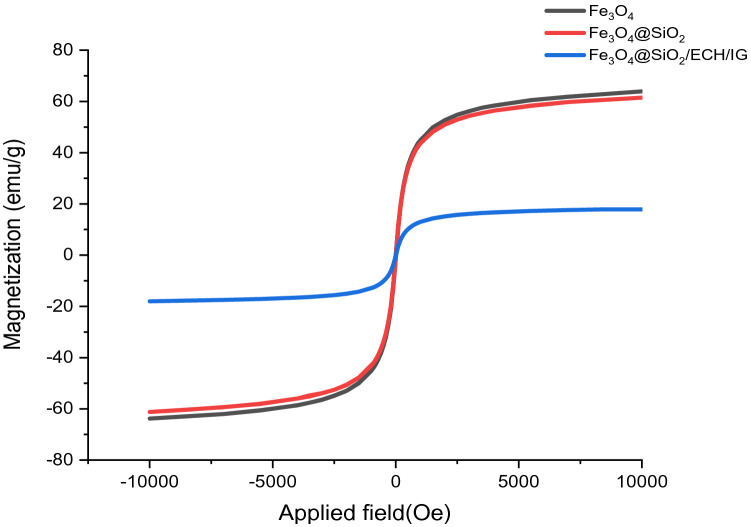
VSM analysis of the prepared Fe_3_O_4_@SiO_2_/ECH/IG.

The magnetic features of Fe_3_O_4_, Fe_3_O_4_@SiO_2_ and Fe_3_O_4_@SiO_2_/ECH/IG were investigated using VSM analysis and the magnetization cycles of the samples are plotted in Fig. 6. As it can be noticed, the particles have zero remanent magnetization so the particles display superparamagnetic behavior. The lack of net magnetization in the absence of an external field allows superparamagnetic nanoparticles to avoid magnetic aggregation^39^. Magnetic hysteresis loop measurements indicated that the maximum saturation magnetization value of Fe_3_O_4_@SiO_2_/ECH/IG (17.162 emus g^−1^) was less than Fe_3_O_4_ (63.9 emus g^−1^) which proved the incorporation of IG on the surface of Fe_3_O_4_ (Fig. 5)^25^.

**Figure 7:**
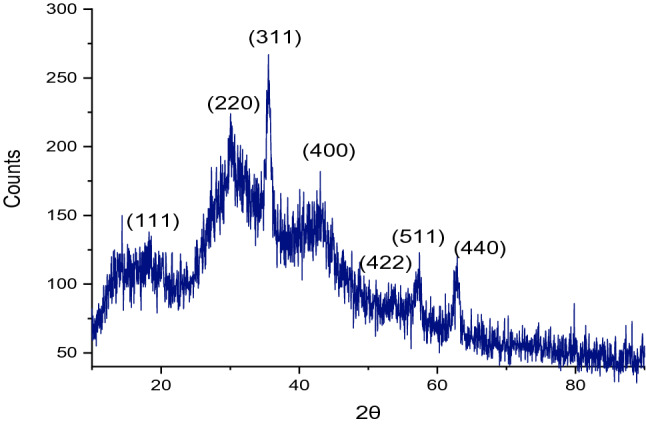
XRD pattern of Fe_3_O_4_@SiO_2_/ECH/IG.

In Fig. 7, the XRD pattern of Fe_3_O_4_@SiO_2_/ECH/IG shows the six characteristic diffraction peaks at 2*θ* 30.064°, 35.452°, 43.038°, 53.547°, 57.168°, and 62.728° corresponding to the (220), (311), (400), (422), (511), and (440) reflection crystal plans of Fe_3_O_4_ respectively (JCPDS card no. 00-001-1111, 00-002-0459). The broad diffraction peak at 2*θ* value 10–20° was attributed to the amorphous structure of isinglass^25^. Another broad diffraction peak around 25–35° indicated the formation of an amorphous SiO_2_ shell around Fe_3_O_4_ (JCPDS card no.00-002-0278). (The reference card numbers were collected from the X'pert HighScore Plus version 1.0d software developed by the PANalytical B.V.).

The specific surface area, pore volume and size analysis were determined by BET methods. According to the BET analysis results shown in Fig. S2 (see supporting information), the adsorption–desorption isotherms display a type IV isotherm with an H_3_ hysteresis loop. The BET surface area is 8.4324 m^2^/g and the adsorption average pore diameter (4 V/A by BET) is 24.49352 nm. The single-point adsorption total pore volume is 0.051635 cm^3^/g (Table S4, see supporting information).

To investigate the thermal stability of the Fe_3_O_4_@SiO_2_/ECH/IG, its thermogravimetric analysis (TGA) was carried out under Ar atmosphere at a temperature varying from 50 to 800 °C. The total weight loss of the nanocatalyst was around 50% (Fig. S3 (see supporting information)). The first weight loss at around 100 °C is attributed to the physically adsorbed water or residual organic solvents in the prepared nanocatalyst. The weight loss at ~ 250 °C continued to ~ 400 °C is related to decomposition of collagen peptide and grafted molecules onto silica surface. The last weight loss from 400 to 800 °C can be ascribed to the combustion of residual coating agents.

### Investigation the catalytic activities of Fe_3_O_4_@SiO_2_/ECH/IG for the synthesis of spirooxindole derivatives 4a-t

**Figure 8:**
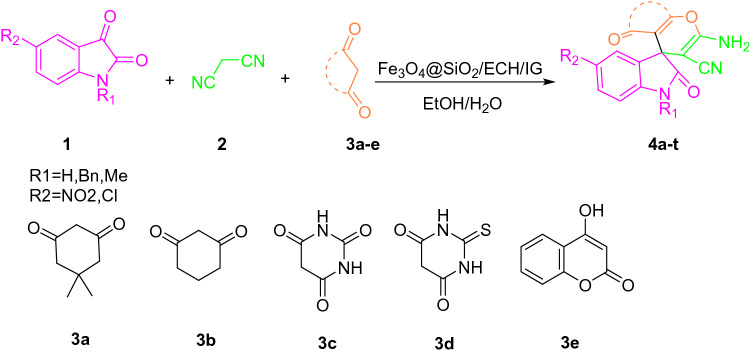
Schematic representation of the Fe_3_O_4_@SiO_2_/ECH/IG and its catalytic activity in the one-pot synthesis of spirooxindole derivatives (4a-t) through multicomponent reaction (MCR) strategy.

The catalytic behavior of Fe_3_O_4_@SiO_2_/ECH/IG was investigated for the synthesis of spirooxindole derivatives via a three- component reaction between CH-acids, malononitrile, and isatin derivatives under different conditions. To find the optimal reaction conditions, various factors such as catalyst loading, solvent, time and reaction temperature were scrutinized in a model reaction including dimedone (**1a**), malononitrile (**2a**), and isatin (**3a**) to estimate the proper catalytic loading and time (Fig. 8). Amid different solvents, the mixture of EtOH/H_2_O (1:1) was completed in a shorter time and gave a better yield (Table S1).

For further optimization, the effect of temperature, type of catalyst, and the amount of catalyst were also investigated and tabulated in Table S2 and S3 (See supporting information). The results revealed the high performance of Fe_3_O_4_@SiO_2_/ECH/IG due to synergistic effects and improved number of active sites on surface. The highest conversion of 94% was reached for 10 mg catalyst loading at 60 °C. Obviously, the increase in catalyst loading was not favorable. On the other hand, with an amount of catalyst of 10 mg, lowering the temperature leads to a decrease in the reaction yield.

To generalize the optimum conditions, different spirooxindole derivatives from 4a-t were prepared through a one pot reaction of isatin derivatives 1, malononitrile 2 and 1,3 dicarbonyl derivatives **3a–3e** in the presence of Fe_3_O_4_@SiO_2_/ECH/IG (Fig. 7). The results are summarized in Table 1. As expected, the presence of electron-withdrawing groups on isatin can enhance the rate and yield of the reaction. The best result in the shortest time was related to the 5-nitro isatin derivative. Contrariwise, the isatin with an electron-donating group provided product with a lower yield.

**Table 1:** Synthesis of spirooxindole derivatives in the presence of Fe_3_O_4_@SiO_2_/ECH/IG.

Entry	R_1_	R_2_	1,3-dicarbonyl compound	Time (min)	Yield (%)	m.p. °C (reported)	Product
1	H	H	**3a**	10	94	301–303 (302–306)^40^	4a
2	H	H	**3b**	10	88	282–284 (278–280)^41^	4b
3	H	H	**3c**	20	81	267–268 (268–270)^41^	4c
4	H	H	**3d**	15	85	230–232 (240–241)^402^	4d
5	H	H	**3e**	25	80	301–303 (290–292)^21^	4e
6	H	NO_2_	**3a**	10	89	> 300 (302–304)^413^	4f.
7	H	NO_2_	**3b**	5	85	> 300 (306–307)^424^	4g
8	H	NO_2_	**3c**	5	93	286–288 (288–289)^435^	4h
9	H	NO_2_	**3d**	10	98	240–242 (253–255)^446^	4i
10	H	NO_2_	**3e**	30	82	297–299 (294–296)^457^	4j
11	H	Cl	**3c**	20	70	234–236 (240–242)^46^	4k
12	H	Cl	**3e**	30	79	> 300 (300–302)^46^	4l
13	Bn	H	**3a**	10	86	284–286 (281–282)^47^	4m
14	Bn	H	**3b**	5	92	282–284 (282–284)^48^	4n
15	Bn	H	**3e**	30	88	273–275 (280–282)^49^	4o
16	Me	H	**3a**	10	80	260–262 (255–258)^50^	4p
17	Me	H	**3b**	10	83	244–246 (243–245)^51^	4q
18	Me	H	**3c**	35	94	279–281 (285–286)^52^	4r
19	Me	H	**3d**	15	92	274–276 (280–282)^53^	4s
20	Me	H	**3e**	30	83	281–283 (283–285)^54^	4t

*Reaction condition: Isatin 1 (1 mmol), 2 (1 mmol), 1,3-dicarbonyl 3 (1 mmol), 10 mg catalyst, and 3 ml solvent at 60 °C.

**Figure 9:**
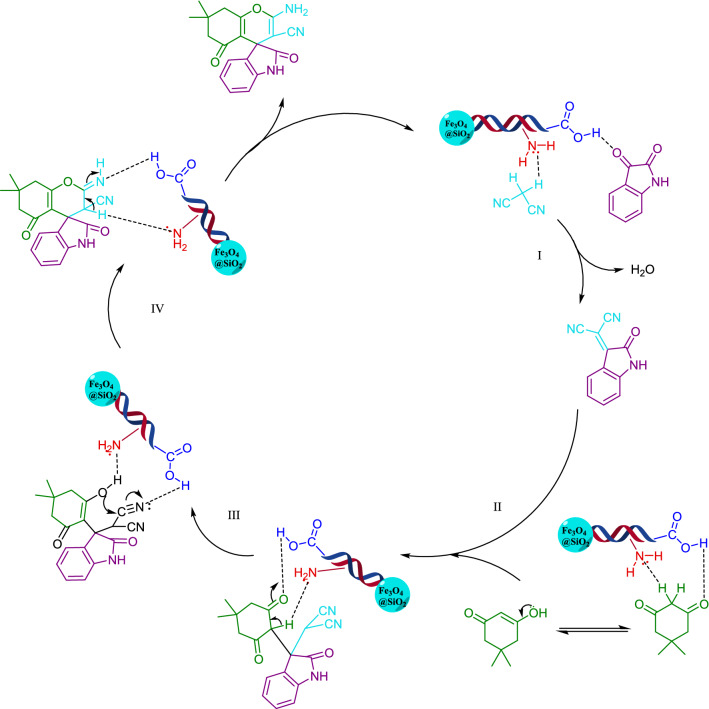
Proposed catalytic mechanism of Fe_3_O_4_@SiO_2_/ECH/IG.

The proposed mechanism of the model reaction for spirooxindole derivative synthesis is mentioned in Fig. 9. In the first step the bifunctional catalyst activated the carbonyl group of isatin by protonation. On the other hand, the amine group of catalyst take the acidic hydrogen of malononitrile. The 1,3 dicarbonyl derivatives was activated and became to enol form through the interaction with functional group of isinglass. The reaction of the first intermediate with activated enol form of dicarbonyl derivatives gives the intermediate (II). Cyclisation, dehydration and tautomerization of imine formed the desire product.

### Catalyst recyclability

The easy separation of Fe_3_O_4_@SiO_2_/ECH/IG heterogenious catalyst was mentioned further. In this regard, the recyclability of the nanocatalyst in the model reaction was investigated. At the end of the reaction, Fe_3_O_4_@SiO_2_/ECH/IG was collected by an external magnetic field and washed with ethanol and water. The dried magnetic nanocatalyst was successively used for four times in the model reaction with a yield as 89%. According to the results displayed in Fig. S4 (see supporting information), there is no significant reduction in the catalytic efficiency of Fe_3_O_4_@SiO_2_/ECH/IG. FTIR spectra of the recycled catalyst were recorded after multiple cycles and compared with the fresh catalyst (Fig. S1). It is clear that the used catalyst has not endure any structural changes.

In order to demonstrate the efficacy of the prepared Fe_3_O_4_@SiO_2_/ECH/IG catalyst, the catalytic activity in the preparation of spirooxindole derivatives was compared with the previous reports. The present catalyst has several advantages in terms of reaction time, solvent, and yield over the reported studies which are tabulated in Table 2.

**Table 2:** Comparison of the present catalyst for the synthesis of spirooxindole derivatives with reported studies.

Entry	Catalyst	Conditions	Time(min)	Yield (%)	References
1	[Bmim]OH	Solvent free/R.T	600	98	^55^
2	Tris-hydroxymethyl aminomethane	Ethanol/R.T	240	94	^56^
3	Trisodium citrate dihydrate	EtOH:Water/R.T	120	96	^57^
4	g-C_3_N_4_/SO_3_H	EtOH:Water/reflux	40	95	^58^
5	Fe_3_O_4_@SiO_2_/ECH/IG	EtOH:Water/60 °C	10	94	This work

### Experimental section

#### Reagents and apparatus

All reagents and materials were purchased from commercial sources and used without purification. All of them were analytical grade. The commercially swim bladders were purchased from grocery store. The known products were identified by comparison of their melting points. Melting points were determined in open capillaries using an Electrothermal 9100 instrument. Infrared (IR) spectra were acquired on a Shimadzu FT-IR-8400S spectrometer with spectroscopic grade KBr. The ^1^HNMR (500 MHz) were obtained on a Bruker Avance DPX-300 instrument. The spectra were obtained in DMSO-d6 relative to TMS as internal standard. Scanning electron microscopy (SEM) was recorded on a VEG2/TESCAN 30kv with gold coating, and energy dispersive X-ray spectroscopy (EDX) was recorded on a VEG//TESCAN-XMU.

### General procedure for the preparation of the Fe_3_O_4_@SiO_2_ NPs

The superparamagnetic iron oxide nanoparticles **(**SPIONs) were synthesized using a co-precipitation method described before^59,60^. Typically, FeCl_3_·6H_2_O (1.215 g) and FeCl_2_·4H_2_O (0.637 g) (Fe^2+^/Fe^3+^ = 1:2) were dissolved in deionized water (20 mL) under an inert atmosphere to get a homogenous solution. Chemical precipitation was carried out by the slow addition of NaOH solution (25%), stirring vigorously at 80 °C for 60 min, until the pH = 10 was attained. The obtained magnetic particles were separated by an external magnetic field, washed three times with deionized water and ethanol (25 mL), and dried in a 65 °C oven for 24 h. After that 1 g of the prepared Fe_3_O_4_ was dispersed in deionized water (50 mL) and stirred for 30 min. Next, a mixture of ammonia (5 ml) and ethanol (50 ml) was added to the flask followed by adding 1.5 ml TEOS. The mixture was stirred for 24 h in room temperature. The prepared Fe_3_O_4_@SiO_2_ was magnetically separated, washed sequentially with water and ethanol and dried in a vacuum oven at 50 °C ^61^.

### General procedure for the preparation of Fe_3_O_4_@SiO_2_/ECH

To prepare Fe_3_O_4_@SiO_2_/ECH nanoparticles, 1 g of the obtained Fe_3_O_4_@SiO_2_ was poured into a round bottom flask containing 3 ml ethanol. Then, ECH (1 ml) was slowly added and the mixture was stirred for 5 h at 60 °C. The resulting precipitate was then magnetically separated, washed with water and ethanol to remove unreacted reagents, and dried in a vacuum oven at 50° C ^62^.

### General procedure for the preparation of Fe_3_O_4_@SiO_2_/ECH/IG

Initially the isinglass was milled to obtain a white powder. 0.1 g of the dried Fe_3_O_4_@SiO_2_ was dissolved in 20 ml ethanol and mixed with 0.2 g of isinglass. The mixture was sonicated for 30 min and then stirred at room temperature for 1 h. finally the obtained precipitate was magnetically separated, washed with water and ethanol and dried at 50 °C.

### General experimental procedure for the synthesis of benzimidazoles derivatives catalysed by Fe_3_O_4_@SiO_2_/ECH/IG

A mixture of isatin (1 mmol), malononitrile (1 mmol), various 1,3 dicarbonyls (1 mmol), and Fe_3_O_4_@SiO_2_/ECH/IG (0.02 g) in EtOH/water (1:1) had been stirred for an appropriate period of time. After completion of the reaction as indicated by TLC, the reaction mixture had been dissolved in hot ethanol and the catalyst was recovered by an external magnet, washed, dried and then reused in successive reaction. The reaction mixture had been recrystallized with ethanol to afford pure desired substituted benzimidazoles^63^.

## Conclusions

In summary, we devised a novel collagen-coated superparamagnetic organic–inorganic hybrid catalyst, Fe_3_O_4_@SiO_2_/ECH/IG, which exhibited radically enhanced catalytic activity in the synthesis of a wide range of substituted spirooxindole derivatives through a one pot atom economical condensation of isatin, dimedone, and malononitrile under mild conditions. This bifunctional heterogeneous catalyst efficiency is achieved in several aspects, such as high product yields under mild conditions, stability, recyclability, and high reaction rate. Furthermore, the easy separation and removal from the reaction environment makes this catalyst a good choice for use in drug synthesis applications. These results affirmed that the novel Fe_3_O_4_@SiO_2_/ECH/IG can be considered as a versatile catalyst for promoting chemical reactions.

## Supplementary Information

Supplementary Information.
