# Supplementary material for: Collagen-coated superparamagnetic iron oxide nanoparticles as a sustainable catalyst for spirooxindole synthesis
Source: Sci Rep. 2022 Apr 12;12:6104. doi: 10.1038/s41598-022-10102-5 (PMC9005729; doi:10.1038/s41598-022-10102-5)
Supplement: Supplementary file 1 — Supplementary Information. [file 41598_2022_10102_MOESM1_ESM.docx]

**Collagen-Coated Superparamagnetic Iron Oxide Nanoparticles as a Sustainable Catalyst for Spirooxindole Synthesis**

Shima Ghanbari^1^, Maryam Esmkhani^1^, Shahrzad Javanshir ^1*^

^1^ Heterocyclic Chemistry Research Laboratory, Department of Chemistry, Iran University of Science and Technology, 16846-13114, Tehran, Iran

[**Figure S 1:** FT-IR spectra of the Fe_3_O_4_@SiO_2_/ECH/IG, Fe_3_O_4_@SiO_2_, Fe_3_O_4_ and reused Fe_3_O_4_@SiO_2_/ECH/IG. 2](#_Toc85660003)

[**Figure S 2:** BET analysis of Fe_3_O_4_@SiO_2_/ECH/IG. 3](#_Toc85660004)

[**Figure S 3:** TGA analysis of Fe_3_O_4_@SiO_2_/ECH/IG. 3](#_Toc85660005)

[**Figure S 4:** Recyclability of Fe_3_O_4_@SiO_2_/ECH/IG 4](#_Toc85660006)

[**Figure S 5:** ^1^HNMR spectrum of the 4i derivative 4](#_Toc85660007)

[**Table S 1**: Effect of solvent on model reaction in the presence of Fe_3_O_4_@SiO_2_/ECH/IG 5](#_Toc85660008)

[**Table S 2:** Investigation of different types of catalyst in model reaction 5](#_Toc85660009)

[**Table S 3:** Investigation the amount of catalyst 5](#_Toc85660010)

**Figure S 1:** FT-IR spectra of the Fe_3_O_4_@SiO_2_/ECH/IG, Fe_3_O_4_@SiO_2_, Fe_3_O_4_ and reused Fe_3_O_4_@SiO_2_/ECH/IG.

**Figure S 2:** BET analysis of Fe_3_O_4_@SiO_2_/ECH/IG.

**Figure S 3:** TGA analysis of Fe_3_O_4_@SiO_2_/ECH/IG.

**Figure S 4:** Recyclability of Fe_3_O_4_@SiO_2_/ECH/IG


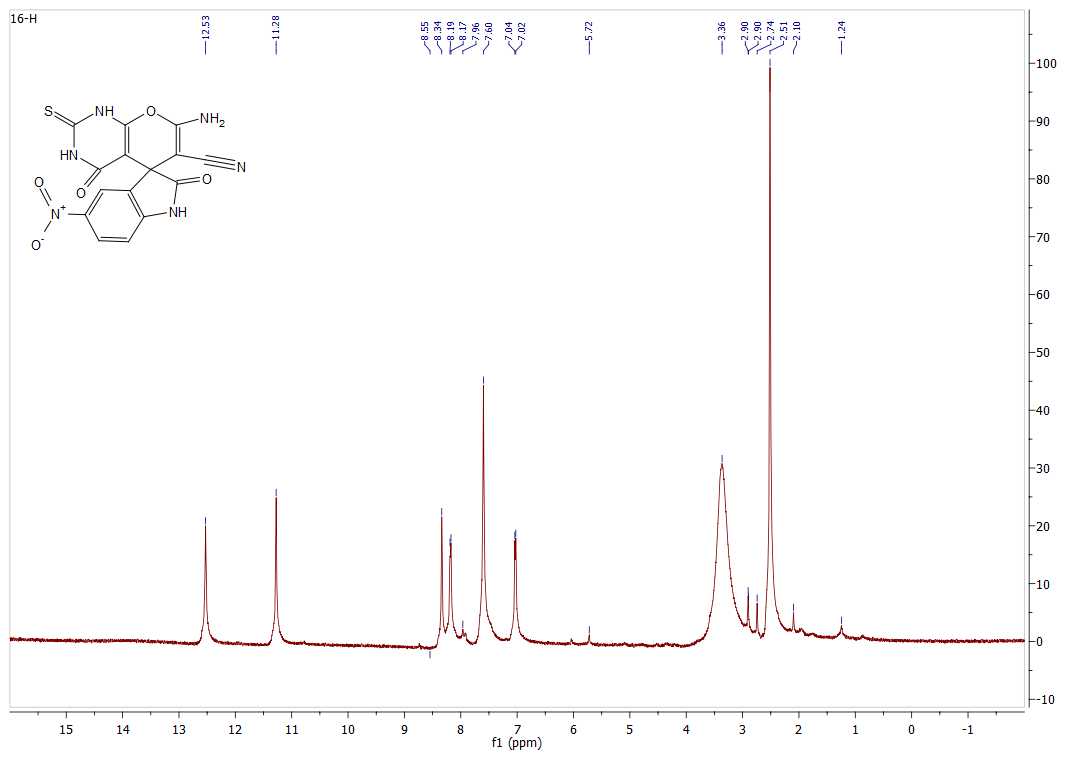


**Figure S 5:** ^1^HNMR spectrum of the 4i derivative

**Table S 1**: Effect of solvent on model reaction in the presence of Fe_3_O_4_@SiO_2_/ECH/IG

| Entry | Solvent | Time(min) | Yield (%) |
| --- | --- | --- | --- |
| 1 | Ethanol | 30 | 92 |
| 2 | water | 30 | 83 |
| 3 | EtOH/water (1:1) | 30 | 94 |
| 4 | CH_3_CN | 30 | 21 |
| 5 | CHCl_3_ | 30 | 17 |
| 6 | MeOH | 30 | 61 |

*Reaction condition: Isatin 1 (1 mmol), 2 (1 mmol), 1,3-dicarbonyl 3 (1 mmol), 10 mg catalyst, and 3 ml solvent at 60 °C.

**Table S 2:** Investigation of different types of catalyst in model reaction

| Entry | Catalyst | (C°)Temperature | (%)yield |
| --- | --- | --- | --- |
| 1 | Fe_3_O_4_@SiO_2_/ECH/IG | rt | Trace |
| 2 | Fe_3_O_4_@SiO_2_/ECH/IG | 40 | 69^a^ |
| 3 | Fe_3_O_4_@SiO_2_/ECH/IG | 60 | 83^a^ |
| 4 | Fe_3_O_4_@SiO_2_/ECH/IG | 80 | 78^a^ |
| 5 | Fe_3_O_4_@SiO_2_/ECH/IG | 60 | 94^a^ |
| 6 | IG | 60 | 91^b^ |
| 7 | Fe_3_O_4_ | 60 | 74^b^ |
| 8 | Fe_3_O_4_@SiO_2_ | 60 | 75^b^ |
| 9 | Fe_3_O_4_@SiO_2_-ECH | 60 | 85^b^ |

*Reaction conditions: isatin (1 mmol), malononitrile (1 mmol), dimedone (1 mmol), catalyst (0.01 g) in EtOH/water (1:1) 3ml

^a^ 10min ^b^ 30min

**Table S 3:** Investigation the amount of catalyst

| Entry | (mg) Catalyst | (%)Yield |
| --- | --- | --- |
| 1 | 3 | 66 |
| 2 | 5 | 79 |
| 3 | 10 | 94 |
| 4 | 15 | 94 |

*Reaction condition: Isatin 1 (1 mmol), 2 (1 mmol), 1,3-dicarbonyl 3 (1 mmol), and 3 ml solvent at 60°C for 30 min.

**Table S4:** Summary of the BET (m^2^/g), pore volume (cm^3^/g), pore size (Å) and surface aspect of Fe_3_O_4_@SiO_2_/ECH/IG

| BET (m^2^/g) | Pore volume | Pore size (Å) |
| --- | --- | --- |
| Surface area at  P/P^o^= 0.298949197: 8.6115 m²/g  m²/g  BET Surface Area: 8.4324 m²/g | Adsorption total pore volume of pores:0.051635 cm³/g | Adsorption average pore diameter  (4V/A by BET): 24.49352 nm |
